# Participatory Epidemiology: Use of Mobile Phones for Community-Based Health Reporting

**DOI:** 10.1371/journal.pmed.1000376

**Published:** 2010-12-07

**Authors:** Clark C. Freifeld, Rumi Chunara, Sumiko R. Mekaru, Emily H. Chan, Taha Kass-Hout, Anahi Ayala Iacucci, John S. Brownstein

**Affiliations:** 1 Children's Hospital Informatics Program at Harvard-Massachusetts Institute of Technology, Division of Health Sciences and Technology, Boston, Massachusetts, United States of America; 2 Division of Emergency Medicine, Children's Hospital Boston, Boston, Massachusetts, United States of America; 3 Department of Epidemiology, Boston University School of Public Health, Boston, Massachusetts, United States of America; 4 Public Health Surveillance Program Office, Office of Surveillance, Epidemiology, & Laboratory Services, Centers for Disease Control and Prevention, Atlanta, Georgia, United States of America; 5 Internews, Nairobi, Kenya; 6 Department of Pediatrics, Harvard Medical School, Boston, Massachusetts, United States of America

## Abstract

Clark Freifeld and colleagues discuss mobile applications, including their own smartphone application, that show promise for health monitoring and information sharing.

**Summary Points**

- Traditional health systems serve a key role in protecting populations, but are typically hierarchical, and information often travels slowly.
- Novel Internet-based collaborative systems can have an important role in gathering information quickly and improving coverage and accessibility.
- Mobile Internet usage is growing rapidly worldwide, making real-time information tools more readily available to both clinicians and the general public.
- We present a brief summary of some promising mobile applications for health monitoring and information sharing, together with preliminary results from a study of our deployment of a smartphone application which enabled the general public to report infectious disease events.
- These early efforts at tapping the power of mobile software tools illustrate potentially important steps in improving health systems as well as engaging the public as participants in the public health process.

In traditional clinical and public health structures, information flows through a hierarchy of providers and local or national authorities, who then communicate with the public via periodic announcements [1]. Meanwhile, broad adoption of the Internet around the world has enabled a new class of participatory systems that allow people to contribute and share information and work together in real time [2]. Wikipedia is perhaps the best-known such project. In the field of public health, online patient communities provide a forum for patients to share their experiences, collect information, and inform biomedical researchers [3]–[5]. Participatory systems in which data and intelligence are gathered from the population, traditionally through discussion or surveys, have also been used to gain an understanding of disease transmission, especially for zoonotic diseases [6]. However, new internet community-based systems represent a departure from the careful control, verification, and data-informed actions of traditional structures, but can provide advantages in scalability, coverage, timeliness, and transparency. Furthermore, engaging the public transforms users from passive recipients of information to active participants in a collaborative community, helping to improve their own health as well as the health of those around them.

The rise in adoption of mobile phones and the Internet, in both industrialized and developing countries, has provided additional opportunities in “crowdsourcing,” which is engaging large groups of people to perform a task [7],[8]. Mobile phones hold particular promise for this type of opportunity because they can be used as point-of-care devices, function in remote locations, and are readily carried and used at any time [9],[10]. In this paper we outline examples of mobile systems for public health, illustrating some of the key concepts, opportunities, and successes made possible through the combination of emerging mobile technologies and user engagement (Table 1). We also detail our own contribution, the Outbreaks Near Me application for iPhone and Android smartphones (For images and further information please see: http://www.healthmap.org/outbreaksnearme/), built on the HealthMap [11],[12] outbreak monitoring platform.

**Table 1:** Overview of selected mobile applications for health.

Organization	Creation Date	Example Deployments and Locations	Summary of Technology(System Description: Technology and Users, Costs, Openness)	Web Site
FrontlineSMS, FrontlineSMS:Medic	2005	Many applications, including health & emergency alerts, as well as pest/disease control. Malawi, Honduras, other developing countries.	• Two-way communication platform via short messaging service (SMS) – only requires mobile phone connection, no Internet, between people whose contact information is known.• Software is open source (no cost to users), each implementation requires one laptop and cellular phone.• Anyone can contribute information by SMS if they know the hub access information (phone number).	http://www.frontlinesms.com http://medic.frontlinesms.com
Ushahidi	2007	Wildlife tracking (Kenya). Tracking medical supply stockouts: Kenya, Uganda, Malawi and Zambia. Disaster response: 4636 project following Port-au-Prince earthquake in January 2010.	• Platform used to collect and visualize crisis data from mobile phones. Data is presented in an online-accessible format.• Software is open source, requires Internet-connected computer (server) for each implementation.• Implementations have used a variety of levels of publicity for contribution information, reaching different populations (e.g. Haiti implementation incorporated widely publicized SMS shortcode number, Twitter hashtags, Web contribution, etc).	http://ushahidi.com
GeoChat	2008	Natural disasters in Thailand, Cambodia and other locations.	• Platform is hosted on the Internet and harnesses Web, email, SMS and Twitter.• Open source software can be downloaded for free or available as a hosted service.• System designed as a group communications technology for use between members of a crisis response team; users have the possibility to contribute through a variety of methods including an SMS gateway (SMS without a mobile connection).	http://instedd.org/geochat
Asthmapolis	2010	Asthma attack and inhaler usage tracking. Currently pilot testing in USA.	• GPS-enabled inhaler coupled with an application for the iPhone, to track and aggregate inhaler usage and location.• Inhaler and mobile diary are not available as of the time of this writing.• Results of patient inhaler use information will be made available to the patient and appropriate physicians and scientists for individual and population surveillance.	http://www.asthmapolis.org
Outbreaks Near Me (HealthMap community)	2009	Infectious diseases – available free to consumers worldwide, generally most popular in developed countries.	• Real-time disease outbreak reporting (from personal experience or official sources).• Applications available for free.• Anyone can download the application and contribute from multiple types of smartphones, data can be viewed by anyone via smartphone or the Web.	http://healthmap.org/outbreaksnearme

## Participatory Mobile Systems for Public Health

The use of mobile systems for health is a growing field with several participatory systems for public health. Selected systems are introduced here; the applications and geographies covered are outlined in Table 1.

One of the earliest efforts, FrontlineSMS (http://www.frontlinesms.com), is a platform for collecting and communicating information via short message service (SMS) [13]. The system is distributed freely (open source), and allows information to be sent and received through a data hub consisting of a laptop and an inexpensive cell phone. Users send “broadcast” messages through this hub to groups of people, including basic forms requesting information, or emergency warning messages, and can also collect the responses via SMS. FrontlineSMS also allows citizens in remote areas to communicate their specific problems and needs directly to health workers who would not otherwise have the capacity to interact with populations in these areas using traditional methods. The system has been used in many countries, and its health-focused spin-off project, FrontlineSMS:Medic (http://medic.frontlinesms.com), is working with partners in Malawi, Burundi, Bangladesh, and Honduras, among others.

Begun in response to postelection violence in Kenya in 2007, Ushahidi (http://ushahidi.com) gained broad recognition and acclaim as an important resource for citizens and responders in the aftermath of the earthquake in Port-au-Prince, Haiti on 12 January 2010 [14]. The system provides an open-source platform for collecting individual reports from users through SMS, Web, and email and provides tools for translating, classifying, and georeferencing these reports; the newest version of the platform further allows for submission via voice message—essential for illiterate users. Aggregated information is presented on a map-based interface accessible via Web and mobile phone. Regarding the Ushahidi deployment in Haiti, Craig Clark of the United States Marine Corps said, “I cannot overemphasize to you what the work of the Ushahidi/Haiti has provided. It is saving lives every day….I say with confidence that there are 100s of these kinds of [success] stories” [15]. Ushahidi has also been deployed in several other countries, including Afghanistan, Uganda, Malawi, and Zambia.

Along the same lines of using SMS communication for situational awareness, GeoChat, one of a suite of open-source software tools designed by InSTEDD (http://instedd.org) [16], aims to achieve faster and more coordinated responses to disease outbreaks and natural disasters. GeoChat enables team members to communicate their position and important information using text messages, email, or a Web browser, with data instantly synchronized on every user's mobile phone or laptop. The system has been successfully launched in Thailand and Cambodia for disease activity monitoring [17]. Although the system does not as yet engage users from the general public, the software is openly available for public use.

Still in its pilot phase, the Asthmapolis (http://asthmapolis.com) project uses mobile devices to enable asthma patients to track asthma attacks. By means of a GPS-enabled inhaler coupled with an iPhone application, users can track the frequency of attacks and where they happen, and the system aggregates data across users to generate a risk map for environmental triggers and improve understanding of the condition. This low-cost system is designed for improved study of underserved populations living with asthma.

## HealthMap and Outbreaks Near Me

Because of its impact across borders and social strata, the 2009 H1N1 influenza pandemic both created broad public awareness of infectious disease threats and presented new challenges for disease detection and response systems. Part of a new generation of online real-time disease outbreak monitoring systems, HealthMap has demonstrated the effectiveness of collecting and filtering news media sources, outside of formal public health channels, for improved situational awareness [18]. However, limitations in coverage, timeliness of reporting, availability of human reviewers, and effectiveness of automated algorithms remain. To address some of these limitations, we created Outbreaks Near Me, where we ask users from the general public to contribute reports from their own knowledge and experiences through a mobile application. We released the Outbreaks Near Me application for iPhone and Android in Fall 2009, during the second wave of pandemic H1N1 infection in the northern hemisphere.

To date, the iPhone application has been downloaded over 110,000 times, and collected over 2,400 submissions from users around the world. Most of these (69%) were approved for publication, and of these, 95% pertained to influenza. We separated the approved submissions into three categories: those referencing a news article (15%); “eyewitness” accounts of local events (41%); and personal accounts of illness, of either the submitter or close associates (13%). Although corroboration or verification of reports is often difficult, our analysts filtered spam, duplicates, and mistakenly submitted reports.

While these reports have proven useful, one particular challenge we have with the system is protecting the privacy of those involved in the event, while still informing the public. Many submissions contain not only a description of the event, but also the user's GPS coordinates or a photograph of the person involved. For these reasons, in addition to classifying and assessing validity, our analysts anonymize the submissions through a three-step process: they first remove identifying features such as names from the text; they then convert the coordinates to a town-level geographic feature name; and finally, place a black bar over pictures to obscure any identifying features. The anonymized record is posted to the public site, but the original submission remains secure in our private hospital database and is revealed only to authorized personnel. While the volume of submissions has not yet been great enough to necessitate automated filtering, one area of future work will be to implement effective automated processing, including anonymization. This type of anonymization has been applied in previous work in this area, for locations [19], medical records [20], and faces in digital images [21].

We took two approaches to evaluating user submissions. First, we constructed a small sample of reports with clearly identifiable events and determined whether the user-submitted information was novel or timely as compared to data from existing HealthMap sources, as shown in Table 2. These examples are actual user submissions from the system, appropriately deidentified through the above process. With outbreaks at schools in particular, several times individuals provided timely information unavailable through other channels.

**Table 2:** A selection of de-identified reports submitted by HealthMap users using the Outbreaks Near Me application.

Date	Category	Location	Excerpt	Info from Pre-existing HealthMap Sources	Comment
20 October 09	Eyewitness	Greendale, Wisconsin	Canterbury elementary school closed until 10/23 due to 30% percent of students out with flu.	None.	This information was also on the school's official Web site.
28 October 09	Personal	Martinsburg, West Virginia	First my 5-year-old son got it then my 18-month-old daughter got it. Now my wife and I both have it [H1N1 influenza].	None.	The submission also includes a report of crowding in the pediatric clinic.
5 September 09	Personal	Lafayette, Louisiana	16-year-old male with undocumented H1N1 with onset 1 week ago. Symptoms of fever to 104 °F, sore throat, body aches, and nonproductive cough for approximately 4 days with Relenza treatment.	None.	Detailed report, most likely coming from a clinician.
14 September 09	Eyewitness	Candia, New Hampshire	3-year-old infected with EEE.	Confirmed in press reports later the same day.	Not all reports were of H1N1. This report was the first EEE case of the New England season.
23 April 09	News-based	Mexico	Canadians returning from Mexico urged to be on alert for flu-like symptoms.	Many media reports immediately following the user submission.	Many media reports of the event were collected through the existing HealthMap system; this submission was among the first indicating international spread.
17 September 09	Eyewitness	Charleston, South Carolina	Three cadets have been placed into quarantine but since then two have returned with no more [flu-like] symptoms.	None.	An H1N1 outbreak at the Citadel Military College was later reported on the school's emergency information Web site.

**Figure 1:**
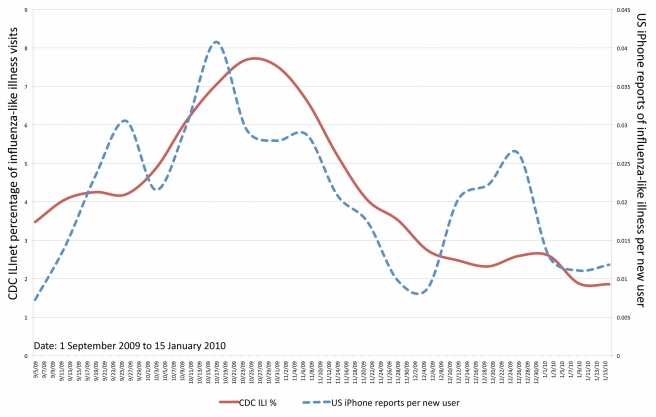
Adjusted volume of H1N1 reports from HealthMap users as compared to weighted influenza-like-illness visit data from the CDC, from September 5, 2009 to January 30, 2010. Pearson's correlation = 0.74, *p*<0.0001.

As a second analysis, we aggregated the submissions and compared their fluctuations in volume over time with the variation in Centers for Disease Control and Prevention (CDC) influenza-like illness (ILI) metrics for sentinel clinical sites in the United States [22]. We computed the number of H1N1 iPhone submissions per application download, to mitigate bias from increased application usage driving increased reporting. Although the result is preliminary and not broadly generalizable given the special circumstances of H1N1, our aggregated metric correlated highly with ILI metrics (Pearson's correlation = 0.74, *p*<0.0001) as shown in Figure 1, suggesting potential use as an additional early indicator for flu activity. Notably, while CDC influenza metrics are generally released at least one week following collection, our metric can be available in near real-time as reports flow into the system.

## Limitations of Crowdsourcing

Despite the potential for participatory epidemiology, many challenges remain. Perhaps the most significant concern is the question of how to corroborate or verify submitted information. Public health officials may rightfully have reservations about this type of data: their obligation to respond to individual reports could represent an added burden to their surveillance responsibilities. However, one preliminary way of analyzing the crowd-sourced data is through cross-validation with other sources, as demonstrated in Table 2. In addition, these systems are by nature venues for two-way information exchange. Rather than simply supplying the end-users with reports, many of the projects we highlighted make use of crowds for evaluating the quality of information as well. By publishing submitted information, they allow users to review and assess the data. This idea is being tested via Ushahidi's Swift River project, amongst others. Swift River further makes use of automated algorithms for scoring and filtering information based on the credibility of sources. Collecting contact information from the person reporting enables system owners to contact the submitter to request additional details if a report raises particular interest. With an effective review and filtering process, we can help avoid information overload.

A related concern with this type of approach is the risk of spreading rumors or a malicious actor gaming the system with false information. To address these concerns many systems require messages to be reviewed by a moderator (either before or after public dissemination), label reports clearly as community contributions, and enable users to provide feedback and even corroboration of submissions, as has proven successful with Wikipedia. Further, because smartphone applications include capability to register GPS coordinates, verification of the proximity of the reporter to the location in question can also be used as a validation tool.

Overall, the “crowdsourcing” approach serves primarily as a complementary new tool, rather than a replacement for either traditional population monitoring efforts or existing new-generation Internet systems.

## Conclusion

Although the data as yet support only preliminary conclusions, we have already seen concrete benefits of community participation in a range of public health settings from pandemics to natural disasters. Promoting technology adoption, verifying reported information, and aligning user incentives remain important challenges for all the systems.

As the processes for collecting and analyzing information from participatory systems become more refined, along with increasing penetration of sophisticated tools such as cellular phones as used in the systems described here, we will see even greater opportunities to gather more detailed structured data for public health reporting. Although some mobile applications rely on expensive smartphones not yet widely available in many resource-poor areas, we believe these phones will rapidly drop in price and see corresponding increases in adoption, as seen with existing mobile technologies. In the future, information from these systems can augment existing public health practice, integrate with clinical tools, and help bring public health services and information to underserved populations. Finally, these early efforts represent an important step in not only improving system outputs but also engaging the public as participants in the public health process.

## Abbreviations

CDC: US Centers for Disease Control and Prevention
ILI: influenza-like illness
SMS: short message service
